# Prevalence of metabolic syndrome in East Azerbaijan-Iran and its determinants factors

**DOI:** 10.34172/jcvtr.2023.31693

**Published:** 2023-12-30

**Authors:** Ali Farshbaf Khalili, Shahryar Razzaghi, Zeinab Nikniaz, Leila Nikniaz, Ali Hossein Zeinalzadeh

**Affiliations:** ^1^Liver and Gastrointestinal Diseases Research Center, Tabriz University of Medical Sciences, Tabriz, Iran; ^2^Tabriz Health Services Management Research Center, Health Management and Safety Promotion Research Institute, Tabriz University of Medical Sciences, Tabriz, Iran; ^3^Social Determinants of Health Research Center, Faculty of Medicine, Tabriz University of Medical Sciences, Tabriz, Iran

**Keywords:** Metabolic syndrome, Prevalence, Cross-sectional study, Risk factor, Iran

## Abstract

**Introduction:**

Metabolic syndrome (MetS) is a prevalent metabolic disorder with increasing prevalence attributed to extended life expectancy. This study aims to investigate MetS prevalence and its determinants in the East-Azerbaijan population.

**Methods:**

Conducted as a cross-sectional study within the East Azerbaijan region, this research is based on a major Lifestyle Promotion Project. The study encompasses 700 participants aged 15 to 65 years, representing the general population and selected using probability proportional to size multistage stratified cluster sampling. MetS diagnoses were conducted using the adult Panel III criteria. Data on socio-demographics, smoking status, and physical activity levels were collected through questionnaires.

**Results:**

Among participants, the mean age was 42.4±12.38 years, and the mean body mass index was 27.69±4.94 kg/m2. The MetS group exhibited higher mean age and body mass index compared to the non-MetS group (*P*<0.001). The prevalence of MetS in the population was 34.2%, with higher rates in females (37.1%) compared to males (30.5%), though this difference wasn’t statistically significant (*P*=0.11). Notably, a substantial distinction was observed between the two groups regarding education levels (*P*<0.001).

**Conclusion:**

The study reveals a significant association between increasing age and higher prevalence of MetS. Furthermore, lower educational levels were linked to an elevated prevalence of MetS. While other socio-demographic factors didn’t demonstrate statistically significant relationships, these findings emphasize the importance of targeted interventions and education in mitigating MetS risks.

## Introduction

 Metabolic syndrome (MetS) encompasses a cluster of metabolic disorders characterized by abdominal obesity, elevated triglyceride (TG) levels, reduced high-density lipoprotein cholesterol (HDL-c), high blood pressure, and increased fasting glucose.^1^ This complex syndrome not only escalates the risk of cardiovascular disease (CVD) but also amplifies the susceptibility to diabetes by fivefold.^2^ The prevalence of MetS varies across different regions; in the United States, it stands at 34.7%, while European countries exhibit rates ranging from 11.6% in Italy to 26.3% in Germany.^3,4^ In Iran, the reported prevalence of MetS is 26%, a moderate level compared to other Asian nations such as Turkey (28.8%), India (31.6%), and China (13%).^5-8^

 Considering the aging population and the concurrent increase in life expectancy in Iran, it is anticipated that the prevalence of MetS will surge in the years ahead.^9^ This necessitates a comprehensive exploration of the risk factors that contribute to and exacerbate the syndrome. Prevailing literature suggests that factors like the adoption of Western dietary patterns, sedentary lifestyles, and improvements in socio-economic status in developing countries might underlie the pandemic of MetS.^10,11^

 While some studies have reported the prevalence and determinant factors of MetS in the Iranian population, it’s essential to recognize the diverse cultural and lifestyle patterns within various regions. This variation underscores the importance of assessing prevalence and determinant factors on a regional basis. Previous research has explored the prevalence of MetS in specific age groups of the population in Tabriz and Basmenj, revealing rates of 55.4% and 12.7%, respectively.^12,13^ However, to date, no study has comprehensively investigated the prevalence of MetS and its interplay with socioeconomic status and lifestyle factors in the population aged 18 and above in Tabriz. Consequently, the objective of the present study is to investigate the prevalence of MetS and discern its determinants within the East-Azerbaijan population.

## Materials and Methods

###  Study Design

 This study was conducted in 2015 as a component of the major Lifestyle Promotion Project (LPP) carried out in the East Azerbaijan province, encompassing both urban and regional areas.

###  Sampling Method

 The sampling method employed was probability proportional to size (PPS) multistage stratified cluster sampling. The specifics of the sampling approach have been previously detailed.^14^ In brief, 150 clusters were selected, with each cluster enrolling five adults, resulting in a total of 750 participants. The research teams conducted household visits as per pre-arranged appointments. After excluding incomplete data, a final sample of 700 participants was subjected to statistical analysis. The study encompasses 700 participants aged 15 to 65 years, representing the general population. The inclusion criteria comprised of being an Iranian citizen, being between the ages of 15 and 65, and having lived in that family for at least six months. The exclusion criteria included having a documented mental disease, as well as cognitive problems, blindness, deafness, and speech disorders.

###  Measurements

 Biochemical Analysis: Fasting blood samples of 10 ml were collected for biochemical analysis. Levels of serum HDL-C, TG, and glucose were measured using enzymatic colorimetric methods. These analyses were performed using a commercially available kit (Pars Azmone, Tehran, Iran) on an automatic analyzer (Abbott, model Alcyon 300, USA) on the same day as the sampling. Blood Pressure Measurement: Blood pressure levels were obtained using a standard manual sphygmomanometer while participants were seated. Waist Circumference (WC) Measurement: WC was measured at the minimum circumference between the iliac crest and the rib cage using an anthropometric tape. Measurements were taken while participants were dressed in light clothing. Classification of MetS: Participants were classified as having MetS based on the criteria defined by the Adult Treatment Panel III.^7^

###  Covariates

 Covariates included age, smoking status, physical activity level, employment status, education level, and body mass index (BMI).

###  Questionnaires

 A questionnaire was administered to obtain socio-demographic information and smoking status. The short form of the International Physical Activity Questionnaire (IPAQ) was employed to assess the level of physical activity.^15,16^

###  Anthropometric Measurements

 Bodyweight was measured using a Seca scale (Dubai, United Arab Emirates) with participants barefoot and wearing light clothing. Height was measured barefoot using a measuring tape, with the subject’s arms hanging freely at their sides. BMI was calculated by dividing weight (kg) by height squared (m^2^).

###  Statistical Analysis

 Statistical analysis was performed using SPSS software version 18. The Kolmogorov-Smirnov test was utilized to assess data normality. Continuous variables were reported as mean and standard deviation (SD), while categorical and nominal variables were reported as frequency and percentage. The independent t-test was employed for between-groups comparisons of continuous variables, while the Chi-square statistical test was used for assessing between-groups comparisons of categorical and nominal variables. Logistic regression models were used to evaluate the risk of MetS based on different demographic factors. Variables exhibiting significant associations in the univariable model were included in the multivariable model. A significance level of 0.05 was adopted, and confidence intervals (CIs) were calculated at the 95% level.

## Results

 In this study, a total of 700 participants were included for analysis. The prevalence of MetS within the study population was found to be 34.2%, with a slightly higher occurrence among females (37.1%) compared to males (30.5%). However, this difference did not reach statistical significance (*P* = 0.11). Additionally, 48% of the participants exhibited elevated waist circumference (WC), and 62% displayed low levels of HDL-C.

Table 1 demonstrates MetS components stratified by gender based on the Adult Treatment Panel III criteria. Demographic characteristics of the participants, stratified by MetS status, are presented in Table 2. The mean age of the participants was 42.4 ± 12.38 years, and the mean body mass index (BMI) was 27.69 ± 4.94 kg/m^2^. Notably, both the mean age and mean BMI were significantly higher in the MetS group compared to the non-MetS group. Furthermore, a significant disparity was observed between the two groups in terms of education level (*P* < 0.001). However, no statistically significant differences were identified between MetS and non-MetS groups with respect to other demographic characteristics.

**Table 1:** metabolic syndrome components stratified by gender based on the Adult Treatment Panel III criteria

**Variables**	**Men (N=288)**		**Women (N=412)**		***P*** ** Value**
	**Mean**	**SD**	**Mean**	**SD**	
Waist circumference ≥ 102 cm in men Waist circumference ≥ 88 cm in women	94.43	13.28	92.12	12.53	0.21*
Triglyceride ≥ 150 mg/dl ^¥^	182.30	119.07	145.16	87.85	< 0.001*
HDL-C < 40 mg/dl in men ^€^ HDL-C < 50 mg/dl in women^€^	39.68	8.29	46.55	9.61	< 0.001*
Systolic blood pressure ≥ 130 mmHg^£^	121.5	18.27	119.89	17.25	0.39*
Diastolic blood pressure ≥ 85 mmHg ^£^	78.52	12.23	77.84	11.19	0.65*
Fasting plasma glucose ≥ 100 mg/dl or under drug treatment for diabetes	90.50	28.23	89.15	31.85	0.06*

* *P* value of independent t-test** ¥** or on drug treatment for elevated triglycerides; **€ **or on drug treatment for reduced HDL-C; **£** or on antihypertensive drug treatment in a patient with a history of hypertension. *P*<0.05 is statistically significant.

**Table 2:** General characteristics of population according to metabolic syndrome status

**Variables**	**Total (N=700)**		**Mets+(N=240)**		**Mets-(N=460)**		***P*** ** value**
	**Mean**	**SD**	**Mean**	**SD**	**Mean**	**SD**	
Age (years)	42.40	12.38	48.47	10.10	39.28	12.28	< 0.001*
BMI (kg/m^2^)	27.69	4.94	30.55	4.55	26.18	4.46	< 0.001^*^
Categorical variables							
	n	%	n	%	n	%	
Sex							
Male	288	41.1	88	36.6	200	43.4	0.11^¥^
Female	412	58.9	153	63.7	260	56.6	
Residency							
Tabriz	341	48.7	124	51.6	216	47	0.3^¥^
Counties	359	51.3	116	48.3	244	53	
Current smoker	78	11.1	22	9.3	54	11.8	
Marriage							
Single	57	8.1	13	5.5	49	10.6	0.05^¥^
Married	643	91.9	227	94.5	411	89.4	
Socioeconomic factors							
Education							
Not educated	103	14.7	57	23.6	46	10.1	< 0.001^¥^
Diploma and lower diploma	501	71.6	161	67	340	73.9	
University	96	13.7	22	9.3	74	16.1	
Employment status							
Employed	288	41.1	92	38.3	196	42.7	0.33^¥^
Not employed	412	58.9	148	61.7	224	57.3	
Physical activity							
Low	249	35.6	83	34.6	165	35.9	0.9^¥^
Medium	214	30.5	75	31.3	139	30.2	
High	237	33.9	82	34.1	156	33.9	

* *P* value of independent t-test; ¥ *P *value of chi-square. *P*<0.05 is statistically significant.

 The outcomes of the univariable and multivariable regression logistic analyses are summarized in Table 3. The results demonstrated a substantial association between age and the odds of MetS (OR = 1.06 [1.04-1.08]; *P* < 0.001). Similarly, BMI exhibited a significant relationship with MetS odds (OR = 1.23 [1.17-1.30]; *P* < 0.001). In terms of education, participants with a diploma (OR = 0.39 [0.22-0.68]; *P* = 0.001) or university education (OR = 0.37 [0.17-0.79]; *P* = 0.01) displayed significantly lower odds of MetS in comparison to those who were illiterate. These associations held true even after adjusting for various covariates in the multivariable model.

**Table 3:** Association between different factors and metabolic syndrome

**Variables**	**Univariate**			Multivariate*		
	**OR**	**95% CI**	***P*** ** value**	**OR**	**95% CI**	***P*** ** value**
Age (years)	1.07	1.05-1.09	< 0.001	1.12	1.04-1.14	< 0.001
BMI (kg/m^2^)	1.23	1.17-1.29	< 0.001	1.31	1.18-1.38	< 0.001
Sex						
Male	1	-	-			
Female	1.34	0.93-1.94	0.11			
Residency						
Tabriz	1	-				
Counties	0.82	0.57-1.18	0.30			
Current smoker	0.76	0.49-1.32	0.38			
Marriage						
Single	1	-	-			
Married	2.04	0.99-4.1	0.05			
Education						
Not educated	1	-	-	1		
diploma	0.38	0.23-0.63	< 0.001	0.40	0.24-0.69	0.001
University	0.24	0.12-0.49	< 0.001	0.36	0.16-0.78	0.01
Employment status						
Not employed	1	-	-			
Employed	0.83	0.57-1.20	0.33			
Physical activity						
Low	1	-	-			
Medium	1.07	0.68-1.68	0.73			
High	1.04	0.67-1.60	0.84			

*Adjusted for variables that have significant association in univariate model *P*<0.05 is statistically significant.

## Discussion

 MetS is not only influenced by genetic factors but is also closely intertwined with various environmental aspects, particularly socio-economic factors. In this study, we aimed to estimate the prevalence of MetS in East-Azerbaijan and explore its associated factors. Our findings revealed a MetS prevalence of 34.2% within our study population. Comparatively, Alizadeh et al. reported a MetS prevalence of 12.7% among individuals aged 18 to 40 years using the ATP III criterion in a Tabriz case-control study conducted in 2013.^13^ Conversely, Froutan et al. found a much higher prevalence of 55.4% in a cross-sectional study among individuals aged 65 to 90 years in Tabriz during the same year.^12^ Meta-analyses have also highlighted varying MetS prevalence rates in the Iranian adult population, with estimates ranging from 26.5% (CI 95% [22.4-30.7]) in Middle Eastern adults to 28% (CI 95% [24-32]) in Iranian adults.^15,16^ These variations are partly attributed to differences in the mean age of participants across studies.

 In this investigation, we delved into the relationship between MetS and various demographic and socio-economic factors. Our results unveiled statistically significant associations between MetS and age, BMI, and education level. Notably, high BMI exhibited a strong correlation with MetS, which is consistent with previous research attributing metabolic disorders to increased BMI.^17,18^ For instance, Mata et al. observed a noteworthy connection between high BMI and MetS in their retrospective study of the Philippine population.^19^ Similarly, Ofer et al. reported that a BMI below 30 carries a predictive value of over 90% for excluding MetS.^20^

 Moreover, our study identified a significant association between lower education levels and increased MetS risk. This finding aligns with prior studies highlighting an inverse relationship between education level and MetS.^1,21-23^ Education level stands as a vital independent determinant, influencing lifestyle, social behaviors, and eating habits.^1^ Educated individuals often enjoy better access to healthier food resources, superior education, and enhanced healthcare services due to their improved socio-economic status.^24^

 Interestingly, our investigation did not reveal a significant relationship between physical activity levels and MetS. This aligns with findings from studies conducted in Korea and the United States.^22,25^ However, differing results have been reported by Suliga et al., where a cross-sectional study on middle-aged Polish populations suggested that low physical activity heightens MetS risk.^18^ This divergence could stem from dissimilar approaches in assessing and classifying physical activity levels.

 Surprisingly, our study did not identify a substantial link between current smoking and MetS. This observation corresponds with the findings of Chichlowska et al who found no significant relationship between smoking and MetS in their prospective cohort study focused on individuals aged 45 to 64 years.^25^ Conversely, Ni et al. indicated that current cigarette smoking could exert a mediating effect on the relationship between socioeconomic factors and MetS in a cross-sectional study.^26^ It’s worth noting that the impact of smoking on health is closely tied to smoking frequency. While investigating the smoking-MetS relationship, accounting for smoking frequency could provide a clearer understanding. In our study, similar to the two aforementioned studies, smoking frequency was not considered.

 While our study’s high sample size and utilization of cluster sampling enhance the generalizability of results to the entire province, the cross-sectional design limits our ability to establish causal relationships. Further longitudinal research could shed light on the causal interplay between the factors examined in this study and MetS.

## Conclusion

 In light of our study’s findings, several key conclusions emerge. Notably, the prevalence of MetS displays a substantial increase with advancing age. Moreover, individuals with lower education levels face a significantly higher risk of MetS compared to their better-educated counterparts. However, among the socioeconomic and demographic factors explored in this study, including gender, marital status, employment status, and residential location (urban or rural), none demonstrated a statistically significant relationship with MetS. Similarly, our investigation did not unveil any significant associations between diverse levels of physical activity and MetS. These findings highlight the complexity of factors influencing MetS prevalence and emphasize the need for comprehensive, multifaceted approaches to tackling this health challenge.

## Acknowledgements

 The authors wish to thank the East Azarbaijan Provincial Health Center, Tabriz Health Services Management Research Center at Tabriz University of Medical Sciences and Eastern Azarbaijan Governor General for their support.

## Competing Interests

 Authors declare that there is no conflict of interest

## Ethical Approval

 Ethical approval for the study was obtained from the Ethics Committee of Tabriz University of Medical Science with the code 1394.383.

## Funding

 East Azarbaijan Provincial Health Center, Tabriz Health Services Management Research Center at Tabriz University of Medical Sciences.
